# Spatial enhancement due to statistical learning tracks the estimated spatial probability

**DOI:** 10.3758/s13414-022-02489-0

**Published:** 2022-04-14

**Authors:** Yuanyuan Zhang, Yihan Yang, Benchi Wang, Jan Theeuwes

**Affiliations:** 1 Key Laboratory of Brain, Cognition and Education Sciences, South China Normal University, Ministry of Education, Guangzhou, China; 2 Institute for Brain Research and Rehabilitation, South China Normal University, Zhongshan Road West 55, Guangzhou, 510000 China; 3 Center for Studies of Psychological Application, South China Normal University, Guangzhou, China; 4 Guangdong Key Laboratory of Mental Health and Cognitive Science, South China Normal University, Guangzhou, China; 5 Department of Experimental and Applied Psychology, Vrije Universiteit, Amsterdam, the Netherlands; 6 Institute Brain and Behavior Amsterdam (iBBA), Amsterdam, the Netherlands; 7 William James Center for Research, ISPA-Instituto Universitario, 1149-041 Lisbon, Portugal

**Keywords:** Attentional capture, Target probability learning, Statistical learning

## Abstract

It is well known that attentional selection is sensitive to the regularities presented in the display. In the current study we employed the additional singleton paradigm and systematically manipulated the probability that the target would be presented in one particular location within the display (probabilities of 30%, 40%, 50%, 60%, 70%, 80%, and 90%). The results showed the higher the target probability, the larger the performance benefit for high- relative to low-probability locations both when a distractor was present and when it was absent. We also showed that when the difference between high- and low-probability conditions was relatively small (30%) participants were not able to learn the contingencies. The distractor presented at a high-probability target location caused more interference than when presented at a low-probability target location. Overall, the results suggest that attentional biases are optimized to the regularities presented in the display tracking the experienced probabilities of the locations that were most likely to contain a target. We argue that this effect is not strategic in nature nor the result of repetition priming. Instead, we assume that through statistical learning the weights within the spatial priority map are adjusted optimally, generating the efficient selection priorities.

## Introduction

Since the 1980, it is well known that visual selective attention can be directed to a nonfixated location in space (e.g., Eriksen & Hoffman, 1973; Hoffman, 1975; Posner et al., 1980; Theeuwes, 1989). The effective use of spatial information is related to the attention mechanism that operates analogous to a beam of light. As a metaphor, Posner (1980) described visual selective attention as a “spotlight that enhances the efficiency of the detection of events within its beam” (p. 172).

In one of their classic experiments, Posner et al. (1978) presented with 80% validity, in the center of the display, an arrow pointing to the location of the upcoming target. This implied that in 80% of trials the arrow pointed to the “valid” (correct) location (i.e., where the target appeared) while in 20% of the trials it pointed to “invalid” location (i.e., at the location opposite to that indicated by the arrow). The results showed that observers were faster and more accurate when the target appeared at the cued location than when it occurred at an un-cued location. Typically, relative to a neutral condition in which no information about the location of the upcoming target is given, there are performance benefits and costs. Even though the classic studies used arrows as cues, similar effects have been found with arbitrary cues such as words “right” or “left” (Vecera & Rizzo, 2004) or an arbitrary number as cue (i.e., 12 means on top of the display; Theeuwes & Van der Burg, 2007).

While some have argued that centrally presented arrows may direct spatial attention in a bottom-up way (Ristic & Kingstone, 2006), this does not hold for arbitrary cues. It is assumed that participants have to interpret the cue and direct spatial attention in a top-down way to the cued location. Some have argued that this type of location cueing in which the cued location varies randomly from trial to trial should be considered as the prime example of knowledge-based, top-down, effortful, trial-to-trial attentional control (see Theeuwes, 2018, for a discussion).

In addition to studies showing top-down location cueing effects (e.g., Posner, 1980), previous studies have already established that the allocation of attention to an area in the visual field depends on target probability. For example, Shaw and Shaw (1977) showed that attention was distributed according to the target location probability. Participants viewed briefly presented displays consisting of eight locations arranged in a virtual circle around the fixation point. The task was to discriminate a single target letter. In two locations, the target location probability was 25%, in four locations it was 10%, and in the remaining two locations it was 5%. This classic study showed that participants allocated attention optimally according to the probability distributions of the target locations. In a study of Geng and Behrmann (2002), participants searched for a target letter among five distractors. Eighty percent of the targets were presented somewhere on one side of the display, while 20% appeared somewhere on the other half side. The results showed that participants allocated attention depending on these target probabilities. Similar findings showing a bias towards particular target locations were reported in other studies (e.g., Fecteau et al., 2009; Ferrante et al., 2018; Geng & Behrmann, 2005; Hoffmann & Kunde, 1999; Jiang et al., 2013; Jiang et al., 2015; Miller, 1988).

There is other evidence indicating that attentional enhancement through statistical learning and top-down attention are different. For example, recently Gao and Theeuwes (2020) manipulated top-down spatial attention and induced attentional enhancement through statistical learning and showed that these effects operated independently. In other words, even when attention was directed in a top-down way to a location in space, participants were even faster when this location was likely to contain a target relatively to other locations. It was concluded that implicit spatial biases due to statistical learning and explicit top-down attention may be different and exert effects on different processes (see Geng & Behrmann, 2005; Stankevich & Geng, 2014, for similar findings).

The current paper investigated in a systematic way how statistical learning of target probabilities affected the allocation of attention in a task in which a singleton distractor was either present or absent. We employed the additional singleton paradigm (Theeuwes, 1991; Theeuwes, 1992) and varied systematically the probability that the target would be presented in one particular location (probabilities of 30%, 40%, 50%, 60%, 70%, 80% and 90%). Because we assume that through statistical learning the weights within the spatial priority map would be adjusted accordingly, we predict that with a higher probability, the facilitation effects would also be stronger. Similarly, if the distractor would happen to be presented at this location, it should cause stronger interference the higher the probability.

## Method

The study was approved by the Ethical Review Committee of South China Normal University (2020-3-013).

### Participants

One hundred and twelve college students between 18 and 26 years of age (10 men and 102 women: with a mean age of 20 years) were recruited from South China Normal University for monetary compensation. They were equally divided into seven groups with different high probabilities of target location (30%, 40%, 50%, 60%, 70%, 80%, and 90%) and each group had 16 participants. Sample size was predetermined based on Lin et al. (2021), which investigated different probabilities of distractor location, and we decided to adopt the same number of participants. They reported normal color vision and normal or corrected-to-normal visual acuity and signed informed consent before the study.

### Apparatus and stimuli

Participants were seated alone in a dimly lit laboratory, 57 cm away from the liquid crystal display (LCD) color monitor with their chin on a chinrest. Stimulus presentation and response registration were controlled by custom scripts written in Python 2.7.

**Fig. 1:**
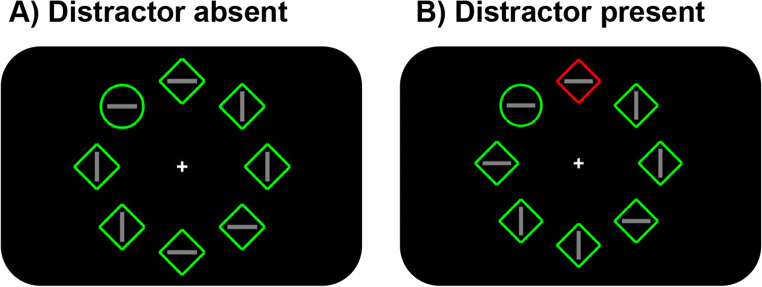
The display setup and possible target and distractor locations for distractor-singleton-absent (**a**) and distractor-singleton-present (**b**) conditions. In this case, the target is green circle and the distractor is red diamond. Participants are asked to indicate the direction of the line segment inside the target. (Color figure online)

The search display consisted of eight discrete stimuli with different shapes (one circle versus seven unfilled diamonds, or vice versa), each containing a vertical or horizontal grey line inside (0.3° × 1.5°; see Fig. 1). These stimuli were presented on an imaginary circle with a radius of 4°, centered at the fixation (a white cross that was visible throughout each trial), against a black background (Red-Green-Blue [RGB]: 0, 0, 0). The circle’s radius was 1°, the unfilled diamond was subtended by 2° × 2°, and each had a red (RGB: 255, 0, 0) or green (RGB: 0, 255, 0) outline.

### Procedure and design

In each trial, a fixation cross appeared in the center of the display and remained visible throughout the trial, and participants were required to fixate at this cross. After 500 ms, the search display was presented for 3000 ms or until response. Participants were asked to search for one circle (target) among seven diamonds, or vice versa, and then to indicate (via button press) the orientation of the line segment inside the target (horizontal = “Left key”; vertical = “Up key”) as fast as possible. If the participant did not respond or if they pressed the wrong key, warning messages were shown. The next trial began after a random intertrial interval (ITI; 500–750 ms).

The target was presented on each trial, and it was equally likely to be a circle or a diamond. Across conditions, two-thirds of the trials were distractor singleton present trials, in which a uniquely colored distractor (i.e., distractor singleton) was presented having the same shape as other distractors, but a different color (red or green with an equal probability). One-thirds of the trials were distractor-singleton-absent trials, in which no distractor singleton was presented. All conditions were randomized within each block.

The target was presented more often in one location (high-probability location) relative to each of other locations (low-probability location) in both distractor-singleton-present and distractor-singleton-absent conditions. For each group, the high-probability location remained the same for each participant and was counterbalanced across participants. This implies that each location in the display was equally likely to be the high-probability location. The participants were not informed about the probability distribution. The distractor singleton appeared at each location with equal chance.

The high probability of target location was 30%, 40%, 50%, 60%, 70%, 80% and 90% for different groups of participants. After a practice block of 20 trials, six experimental blocks (120 trials each) were run for different groups. After performing the search task, we tested participants’ awareness regarding the high-probability target location in the groups with the overall probability of 40%, 50%, 70%, 80%, and 90%. They had to answer three questions: (1) They need indicate whether they were aware that the target was presented more often in one particular location. (2) If they answered with “yes,” they had to indicate which location was high-probability location; if they answered with “no,” they had to guess the high-probability location. (3) They were asked to indicate the confidence about their answer on a 7-points scale (ranging from *not confident at all*, i.e., 0% sure, to *very confident*, i.e., 100% sure).

To evaluate the strength of the evidence for the alternative hypothesis (H1) over the null hypothesis (H0), we calculated the Bayes factor (BF_10_) using Bayesian hypothesis testing (Andraszewicz et al., 2015; Wagenmakers (2018). According to this framework a BF_10_ below 1 reflects evidence in support of H0 such that a BF_10_ of 1–0.33 reflects anecdotal evidence, 0.33–0.1 moderate evidence, and <0.1 strong evidence in favor of the null hypothesis.

## Results

Incorrect trials, no-response trials, and trials on which the response times (RTs) were larger or smaller than 2.5 standard deviations from the average RTs per participant were excluded from analyses across all experiments. Only a small proportion of trials for different groups were excluded: 0.51%, 0.36%, 0.27%, 0.39%, 0.57%, 0.43%, and 0.36% trials were excluded for no response; 2.34%, 2.41%, 2.66%, 2.40%, 2.26%, 2.09%, and 2.67% trials were excluded for RT outliers, and 3.54%, 3.73%, 4.08%, 3.24%, 3.56%, 3.26%, and 2.68% trials were excluded for incorrect responses, for group one (with the high probability of 30%) to seven (with the high probability of 90%), respectively.

### Attentional capture effect

The mean response times (RTs) and mean error rates for different groups (with different overall probabilities of the high-probability target location) are presented in Table 1, respectively. With distractor condition (distractor present vs. absent) as a within-subjects factor and overall probability (30%, 40%, 50%, 60%, 70%, 80%, and 90%) as a between-subjects factor, a mixed ANOVA on mean RTs revealed significant main effects for distractor condition, *F*(1, 105) = 507.06, *p* < .001, η2 P = 0.83, and overall probability, *F*(6, 105) = 2.53, *p* = .025, η2 P = 0.13, but not an interaction, *F*(6, 105) = 1.09, *p* = .371, η2 P = 0.06, BF_10_ = 0.11. It indicates that participants were captured by the distractor singleton regardless of the overall probability of the high-probability target location. The higher the target probability, the faster the response.

**Table 1:** The mean response times (RTs) and mean error rates between distractor-present and distractor-absent conditions for different overall probabilities

Overall probability	Mean RTs (ms)		Mean error rates	
	Distractor present	Distractor absent	Distractor present	Distractor absent
30%	1,318	1,208	0.04	0.02
40%	1,252	1,155	0.04	0.02
50%	1,124	1,035	0.05	0.03
60%	1,247	1,138	0.04	0.03
70%	1,224	1,128	0.04	0.03
80%	1,218	1,129	0.04	0.02
90%	1,111	1,034	0.03	0.02

Similarly, a mixed ANOVA on mean error rates revealed a significant main effect for distractor condition, *F*(1, 105) = 75.27, *p* < .001, η2 P = 0.42, but not for the overall probability, *F*(6, 105) = 0.52, *p* = .794, η2 P = 0.03, BF_10_ = 0.12, nor for interaction, *F*(6, 105) = 1.17, *p* = .329, η2 P = 0.06, BF_10_ = 0.18.

### Learning effect

**Fig. 2:**
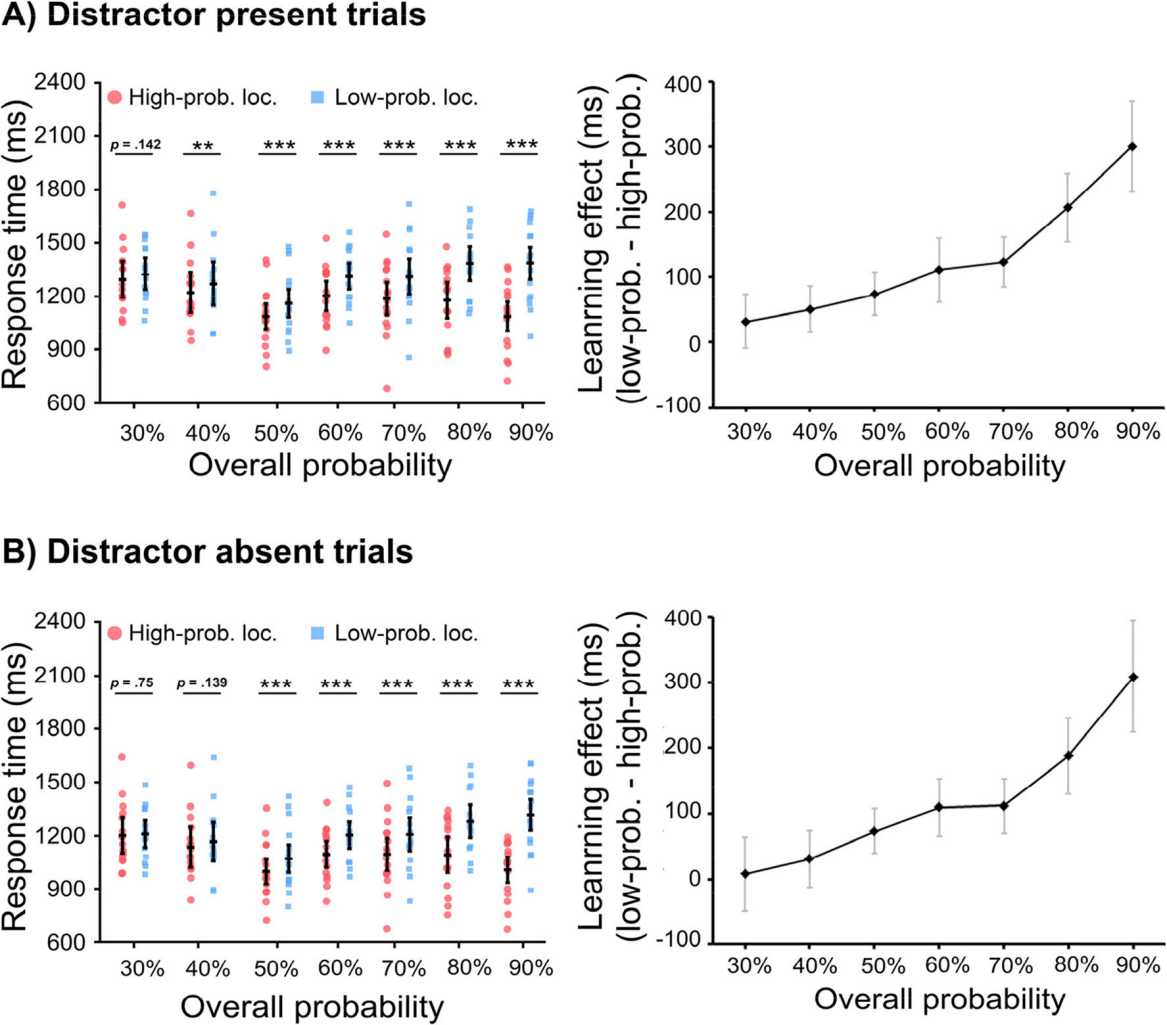
The mean response times (RTs) as a function of the target location and the learning effect (i.e., mean RTs in low-probability target location minus that in high-probability target location) in the distractor-present (**a**) and distractor-absent (**b**) trials. Error bars indicate 95% CIs

The mean RTs for high- and low-probability target location and the difference between them in different distractor conditions are presented in Fig. 2.

#### Distractor-present trials

With target location (high-probability vs. low-probability location) as a within-subjects factor and overall probability (30%, 40%, 50%, 60%, 70%, 80%, and 90%) as a between-subjects factor, a mixed ANOVA on mean RTs revealed a significant main effect for target location, *F*(1, 105) = 251.8, *p* < .001, η2 P = 0.71, but not for overall probability, *F*(6, 105) = 1.75, *p* = .116, η2 P = 0.09, BF_10_ = 0.74. However, a significant interaction was observed, *F*(6, 105) = 19.81, *p* < .001, η2 P = 0.53. That is, the learning effect became larger when the overall probability was higher (as illustrated in Fig. 2a, right panel). Planned comparisons showed that, only in the groups with the overall low probability of 30%, there was no statistical difference between high- and low-probability target location, *t*(15) = 1.55, *p* = .142, Cohen’s d = 0.39, BF_10_ = 0.69; while for other groups, the mean RTs was smaller for high-probability target location than low-probability target location, all *t*s > 3.2, *p*s < .006, Cohen’s *d*s > 0.8 (see Fig. 2a, left panel).

To investigate how the learning effect (reflected by the difference between high-probability and low-probability target location) systematically changed with different overall probabilities, we calculated the learning effect for further analysis by using mean RTs for low-probability target location minus that for high-probability target location. We have attached the results for those comparisons on learning effect between different overall probabilities in Table 2 in the Appendix.

#### Distractor-absent trials

With target location (high- vs. low-probability location) as a within-subjects factor and overall probability (30%, 40%, 50%, 60%, 70%, 80%, and 90%) as a between-subjects factor, a mixed ANOVA on mean RTs revealed a significant main effect for target location, *F*(1, 105) = 160.01, *p* < .001, η2 P = 0.6, but not for overall probability, *F*(6, 105) = 1.71, *p* = .127, η2 P = 0.09, BF_10_ = 0.6. Again, a significant interaction was observed, *F*(6, 105) = 17.11, *p* < .001, η2 P = 0.49. That is, the learning effect became larger when the overall probability was higher (as illustrated in Fig. 2b right panel and Table 2 in the Appendix). Planned comparisons showed that, in the groups with the low probability (i.e., 30% and 40%), there was no statistical difference between high- and low-probability target location, *t*(15) = 0.33, *p* = .75, Cohen’s *d* = 0.08, BF_10_ = 0.27, and *t*(15) = 1.56, *p* = .139, Cohen’s *d* = 0.39, BF_10_ = 0.7, respectively; while for other groups, the mean RTs was smaller for high-probability target location than low-probability target location, all *t*s > 4.9, *p*s < .001, Cohen’s *d*s > 1.24 (see Fig. 2b, left panel).

Furthermore, we also checked the results for mean error rates (see Table 3 in the Appendix) and found that the current results cannot be explained by condition-specific speed–accuracy trade-off.

### Learning over time

To determine whether the target-related learning changed over time, we conducted mixed ANOVAs with experimental block (Block 1, Block 2, Block 3, Block 4, Block 5, Block 6) as a within-subjects factor and overall probability (30%, 40%, 50%, 60%, 70%, 80%, and 90%) as a between-subjects factor for distractor-present and distractor-absent conditions, and for the combination of those two conditions separately.

**Fig. 3:**
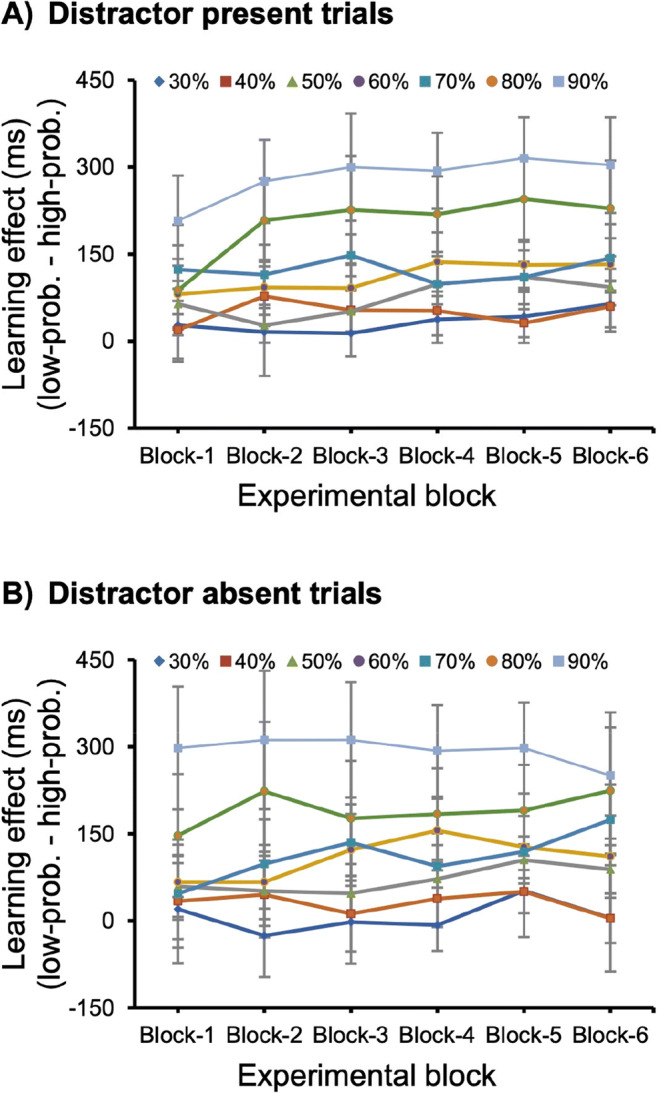
The learning effect for different blocks and different overall probabilities in the distractor-present trials (**a**), distractor-absent trials (**b**). Error bars indicate 95% CIs. (Color figure online)

In the distractor present condition, the results revealed significant main effects for overall probability, *F*(6, 105) = 18.55, *p* < .001, η2 P = 0.52, and experimental block, *F*(5, 525) = 5.62, *p* < .001, η2 P = 0.05, but not an interaction, *F*(30, 525) = 1.29, *p* = .140, η2 P = 0.07, BF_10_ = 0.06 (see Fig. 3a). In the distractor absent condition, there was a significant main effect for the overall probability, *F*(6, 105) = 17.32, *p* < .001, η2 P = 0.5, but not for experimental block, *F*(5, 525) = 1.2, *p* = .31, η2 P = 0.01, BF_10_ = 0.01, nor the interaction, *F*(30, 525) = 0.84, *p* = .718, η2 P = 0.05, BF_10_ < 0.01 (see Fig. 3b). Altogether, these findings indicate that learning was completed after one block being exposed to the regularities, even though for the higher probabilities (80% and 90%) performance seem to asymptote after three blocks of exposure to the regularities.

### Distractor at the high-probability target location

**Fig. 4:**
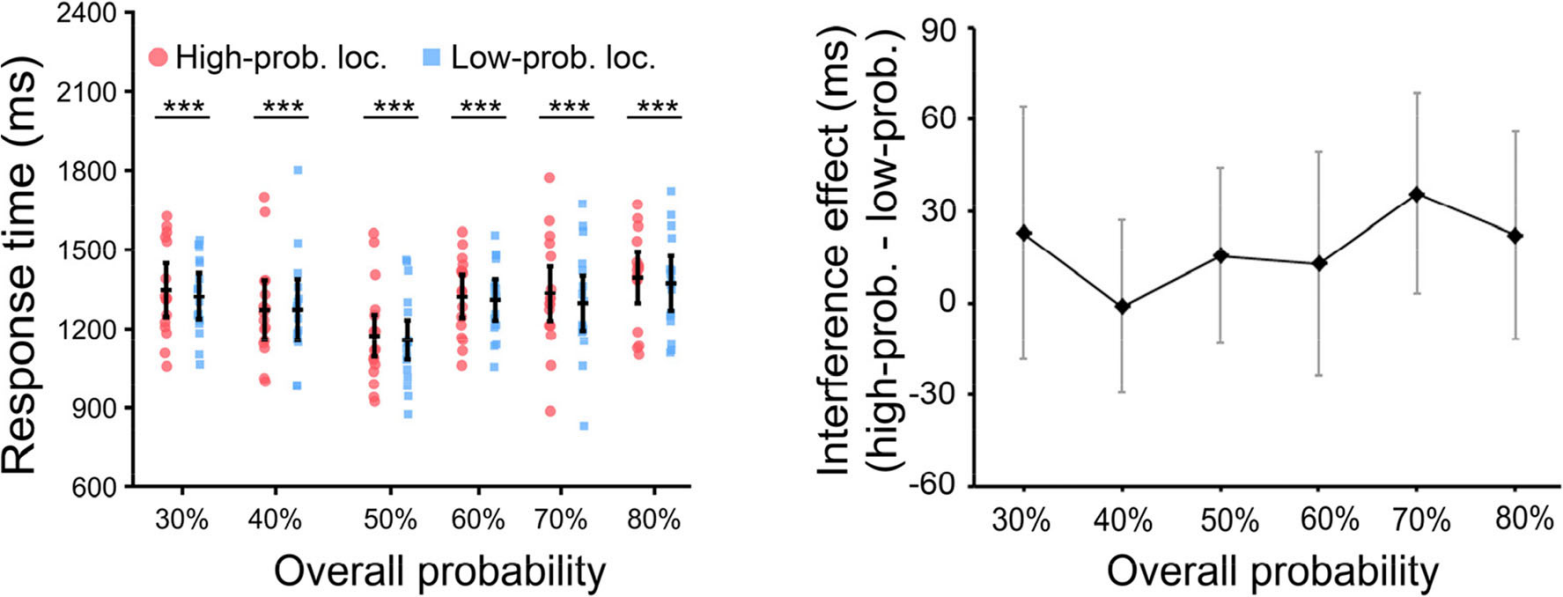
The mean RTs as a function of the *distractor position* (left panel) and the interference effect of distractors (right panel, i.e., mean RTs in high-probability target location minus that in low-probability target location) in the low-probability target condition in the distractor-present trials. Error bars indicate 95% CIs. (Color figure online)

As we assume that through statistical learning the target location is enhanced matching the target probability, we expect that if the distractor happens to be presented at that high-probability location, the interference it causes should also reflect this enhancement in the low-probability target condition. To answer this question, we conducted mixed ANOVAs with distractor position (high- vs. low-probability target location) as a within-subjects factor and overall probability (30%, 40%, 50%, 60%, 70%, 80%)^1^ as a between-subjects factor. The results showed that there were significant main effects for distractor position, *F*(1, 90) = 9.47, *p* = .003, η2 P = 0.1, and overall probability, *F*(5, 90) = 2.72, *p* = .025, η2 P = 0.13. Importantly, there was no significant interaction, *F*(5, 90) = 0.73, *p* = .602, η2 P = 0.04, BF_10_ = 0.08, see Fig. 4.

### Intertrial priming

We speculated that the learning effect could be partially contributed to by the short-lived intertrial location priming (e.g., Maljkovic & Nakayama, 1994), especially for relatively higher overall probabilities, because the manipulation of the probability of target location would generate more location repetitions for the high-probability target location compared with the low-probability target location. To examine this, we compared trials in which the target location of a given trial was identical to the previous trial with trials in which the target location had changed (i.e., repeated vs. nonrepeated). A mixed ANOVA, with repeat condition (repeated vs. nonrepeated) as a within-subjects factor and overall probability (30%, 40%, 50%, 60%, 70%, 80%, and 90%) as a between-subjects factor, showed significant main effects for repeat condition, *F*(1, 105) = 51.49, *p* = < .001, η2 P = 0.33, and overall probability, *F*(6, 105) = 2.2, *p* = .049, η2 P = 0.11, and an interaction, *F*(6, 105) = 16.04, *p* < .001, η2 P = 0.48. Planned comparisons showed that, when the overall probability was 30%, the priming effect showed a trend yet it was not statistically reliable *t*(15) = 1.99, *p* = .066, Cohen’s *d* = 0.5, BF_10_ = 1.22. Note that, however, we did not observe any learning effect in this condition. In the groups with the overall probability of 40%, 50%, and 60%, the priming effect was not statistically reliable, all *t*s < 1.6, *p*s > .131, Cohen’s *d*s < 0.4, BF_10_ < 0.73. For the other groups however, mean RTs were faster for repeated than for nonrepeated target location, all *t*s > 2.89, *p*s < .05, Cohen’s *d*s > 0.72. The results suggest that inter-trial location priming plays no role in case of the lower target probabilities (i.e., 30%, 40%, 50%, and 60%); however, at the higher probabilities (i.e., 70%, 80%, and 90%) location priming adds to the observed effects. This is consistent with our results that the learning effect did not change linearly, instead had a knot point appeared around ~70% estimated by spline model (Cudeck & Klebe, 2002; Geng et al., 2017). That is, the learning effect increased quickly after 70% probability (see Fig. 2).

**Fig. 5:**
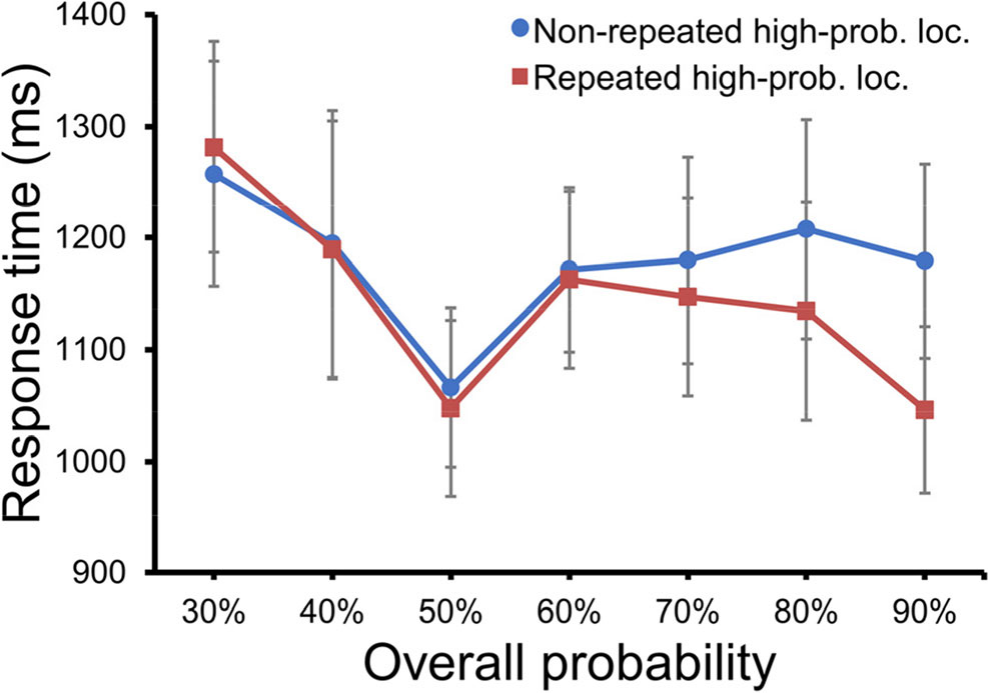
The mean RTs for repeated and non-repeated high-probability locations as a function of the overall probability. Error bars indicate 95% CIs

Although the priming likely contributed to the learning effect observed at the higher probabilities (i.e., 70%, 80%, and 90%), it is important to determine whether priming plays a critical factor. To control for priming, we conducted a linear mixed-model (LMM) analysis (see Huang, Theeuwes, & Donk, 2021a). Mean RTs were entered into the LMMs as a dependent variable, with overall probability as the fixed effect of interest (70%, 80%, 90%; dummy coded). Target location (coded as 1 = high-probability location, 0 = low-probability location) and repeat condition (1 = repeated high-probability location, 0 = nonrepeated high-probability location; dummy coded) were selected as the other fixed effects. By-participants random intercepts were included as the random effect. For comparisons within factors, the degrees of freedom were estimated by Satterthwaite approximation, and the *p* values were obtained from the lmerTest package (Kuznetsova et al., 2017). The estimate (β) of the fixed effect was provided as the measure of the effect size. The results showed a significant fixed effect for target location, χ^2^(1) = 34.83, *p* < .001, observers’ response was faster when the target was located at the high-probability location than at the low-probability location (β = 174.8, *SE* = 28.06), *t*(165.04) = 6.23, *p* < .001. It suggests that even at the higher probabilities (i.e., 70%, 80%, and 90%) the learning effect observed stayed in place when controlling for effect of location priming.

### Awareness analysis

After the experiments, we also checked whether participants were aware of the high-probability target location. However, we only collected data for the groups with the overall probability of 40%, 50%, 70%, 80%, and 90%. Of these groups, respectively 68.8%, 87.5%, 100%, 88%, and 94% participants correctly identified the high-probability target location. This implies that a small number of participants (*n* = 10) can be labeled as unaware of the high-probability location. To examine whether the observed learning effect is also found when participants are not aware, we compared high- and low-probability location for this unaware group regardless of overall probabilities, and observed a significant difference (70 ms), *t*(9) = 2.49, *p* = .034, Cohen’s *d* = 0.79. Although such difference is smaller compared to the aware group (159 ms), *t*(78) = 2.12, *p* = .037, Cohen’s *d* = 0.72, it suggests that being aware of the high-probability target location is not necessary for obtaining the learning effects.

## General discussion

The results of the present study are clear. Participants learned the probabilities of the target regularities present in the display and distributed the attentional resources optimally to obtain the most efficient selection biases. We assume that these biases are optimized by changing the weights within the spatial priority map. We found (1) the higher the target probability, the larger the difference in RT between high- and low-probability conditions for distractor-present and distractor-absent conditions. (2) When the distractor happened to be present at the high-probability location, it caused more interference than when present at a low-probability location. (3) The results suggest that when the difference between high- and low-probability conditions was relatively small (30%), participants were not able to learn the contingencies, and no benefit for the high-probability location was found. (4) Learning was very fast and the effect on target prioritization was already present during the first block.

The current findings suggest that through statistical learning attentional resources are optimally adjusted to obtain the most efficient selection biases. We assume that the weights within the spatial priority map are altered optimally matching the target probabilities present in the display (Ferrante et al., 2018). These findings are very similar to a study by Lin et al. (2021), who showed that distractor suppression also followed the probability distribution of distractors in the display (see also Sauter et al., 2019). The altered weights within the priority map resulted in performance that is tuned optimally to the distractor probabilities in Lin et al. (2021). A recent study by Huang et al. (2021a, b) showed that these weights within the priority map were proactively enhanced, suggesting that the location is already prioritize before display onset. In Huang et al. (2021a, b), study participants performed an additional singleton task on most trials; yet on a subset of trials, participants performed a probe task, in which they had to detect the offset of a probe dot. This probe task was presented before the search display making it possible to examine the distribution of attention before the display was presented. The results showed that a probe dot at the enhanced target location was detected faster than a probe at another location indicating spatial enhancement before the actual search display was presented.

It is important to note that the current findings cannot be explained by intertrial location priming alone (Maljkovic & Nakayama, 1994). The results (combining with the LLM analysis) indicate that at the higher target probabilities (70%, 80%, 90%) location priming did not determine, instead only partially contributed to, the observed effect; yet at the lower target probabilities, repeat trials were not faster than nonrepeat trials.

As in our previous studies, all learning took place in the first block, demonstrating that learning was extremely fast. Lin et al. (2021) reported the same with respect to distractor suppression. Similarly, Wang and Theeuwes (2020) showed that participants not only learn the contingencies fast but that they also adapt learning once the contingencies changes during the course of an experiment.

Consistent with earlier findings, the present experiment demonstrates that the probability of the target appearing at a particular location has a robust effect on attentional selection (e.g., Fecteau et al., 2009; Ferrante et al., 2018; Geng & Behrmann, 2005; Hoffmann & Kunde, 1999; Jiang et al., 2013; Jiang et al., 2015; Miller, 1988). Critically, we show that the attentional benefits scale with the target probabilities. Because most participants were aware of the regularities present in the display we cannot exclude the possibility that the current findings can be explained by top-down, knowledge-based orienting similar to attentional orienting in a Posner-like cueing task. However, it is more likely that the attentional benefits we observed here are not strategic in nature but due to statistical learning of the regularities present in the display, similar to the way we explained suppression of distractor locations due to statistical learning (Lin et al., 2021; Wang & Theeuwes, 2018a, 2018b). This claim is consistent with the finding that those participants that were not aware of the regularities also showed a similar benefit of attentional enhancement of the target location.

We interpret the current findings in terms of the adjustment of weights within the spatial priority map such that the higher the probability that a target is presented at a location the more attentional resources are directed to that location. Previous research has shown that directing attention to a location improves sensitivity. For example, in a signal detection study, Theeuwes and Van der Burg (2007) showed that perceptual sensitivity is increased for detecting targets presented at cued locations relatively to targets presented at uncued locations (see also Ciaramitaro et al., 2001).

It is important to note that when the distractor is presented at the high-probability location it causes interference. Critically however the amount of interference is not related to the target probability. Indeed, Fig. 4 shows that across all target probabilities, the interference caused by the distractor, when it happens to be presented at that location, remains about the same. Given claims that the weights within the spatial priority map are weighted according to the target probabilities, one would expect that with increased weight also the interference caused by a distractor presented at this location would increase. Even this may seem plausible, this is not necessarily the case. When the distractor is presented at a high-probability location (Fig. 4), the target has to be presented at a low-probability location. This implies that attention needs to be disengaged from this high-probability location (that contains a distractor) and shifted towards the target location. Our results suggest that the disengagement of attention from the high probability location is about equally fast irrespective of the attentional weights that are allocated to this location. With more attentional resources allocated to one location there may be fewer resources available for detecting the target at the low-probability location which would imply that the interference effect should get larger with higher probabilities. However, if more resources are allocated to the high-probability target location, and a distractor is presented there, disengagement from that location may be faster. Even though this is very speculative, it may suggest that these effects (fewer resources to find the target, and faster disengagement from the distractor), cancel each other out, resulting the same interference effect across the probabilities.

Our analysis regarding the speed of learning indicated no reliable effect for block in the distractor absent trials while in the distractor present trials it was reliable and did not interact with the target probability. Even though this may suggest that the presence of a distractor slowed learning, this explanation is unlikely because distractor present and absent trials were mixed within blocks. If learning occurs for one type of trials (distractor absent trials) there is no reason to assume that it does not occur for the other type of trials that were mixed within the same block. Rather than assuming that the presence of a distractor slows learning it is more likely that over blocks participants learn to disengage attention faster from the distractor. This may result in a learning effect for distractor present trials only, and it is not affected by the probability manipulation.

In sum, the current study shows that attentional biases are optimized to the regularities present in the display tracking the experienced probabilities of the locations that is most likely to contain a target. We argue that this effect is not strategic in nature nor the result of repetition priming. We claim that through statistical learning attentional resources are optimally adjusted to obtain the most efficient selection biases. We assume that these biases are optimized by changing the weights within the spatial priority map.

## Appendix

**Table 2:** Paired comparisons on learning effect between different overall probabilities for the distractor-present and distractor-absent trials, and the combination of them

Comparisons	Distractor present			Distractor absent		
	*t*(30)	*p*	Cohen’s *d*	*t*(30)	*p*	Cohen’s *d*
30% vs. 40%	0.73	.469	0.26	0.75	.469	0.26
30% vs. 50%	1.67	.106	0.59	2.32	.106	0.82
30% vs. 60%	2.88	.007	1.02	3.31	.007	1.17
30% vs. 70%	3.68	<.001	1.30	3.73	<.001	1.32
30% vs. 80%	5.52	<.001	1.95	3.73	<.001	1.32
30% vs. 90%	6.77	<.001	2.39	6.18	<.001	2.18
40% vs. 50%	1.07	.292	0.38	1.70	.292	0.60
40% vs. 60%	2.49	.019	0.88	2.85	.019	1.01
40% vs. 70%	3.42	.002	1.21	3.32	.002	1.17
40% vs. 80%	5.39	<.001	1.91	3.32	<.001	1.17
40% vs. 90%	6.66	<.001	2.35	5.93	<.001	2.10
50% vs. 60%	1.53	.136	0.54	1.51	.136	0.53
50% vs. 70%	2.34	.026	0.83	1.91	.026	0.68
50% vs. 80%	4.61	<.001	1.63	1.91	<.001	0.68
50% vs. 90%	6.05	<.001	2.14	5.26	<.001	1.86
60% vs. 70%	0.52	.610	0.18	0.11	.610	0.04
60% vs. 80%	3.12	.004	1.10	0.11	.004	0.04
60% vs. 90%	4.88	<.001	1.72	4.30	<.001	1.52
70% vs. 80%	2.96	.006	1.05	4.41	.006	1.56
70% vs. 90%	4.79	<.001	1.70	0.00	<.001	0.00
80% vs. 90%	2.24	.033	0.79	4.41	.033	1.56

**Table 3:** Mean error rates between high-probability and low-probability target location in different overall probabilities for the distractor-present and distractor-absent trials

Overall probability	Distractor present		Distractor absent	
	High prob.	Low prob.	High prob.	Low prob.
30%	0.04	0.04	0.03	0.02
40%	0.04	0.04	0.02	0.03
50%	0.04	0.05	0.03	0.03
60%	0.03	0.04	0.02	0.03
70%	0.03	0.06	0.02	0.04
80%	0.03	0.07	0.02	0.05
90%	0.02	0.11	0.01	0.08

## Footnotes

1 For the 90% group, to make sure that the distractor was equally likely to be present at each location, the distractor was only present at the high-probability location when the target was at the low-probability location. Thus, this group had no trials for low-probability condition in the following analysis.

## References

[CR1] Andraszewicz S, Scheibehenne B, Rieskamp J, Grasman R, Verhagen J, Wagenmakers EJ (2015). An introduction to Bayesian hypothesis testing for management research. Journal of Management.

[CR2] Ciaramitaro VM, Cameron EL, Glimcher PW (2001). Stimulus probability directs spatial attention: An enhancement of sensitivity in humans and monkeys. Vision Research.

[CR3] Cudeck R, Klebe KJ (2002). Multiphase mixed-effects models for repeated measures data. Psychological Methods.

[CR4] Eriksen CW, Hoffman JE (1973). The extent of processing of noise elements during selective encoding from visual displays. Perception & Psychophysics.

[CR5] Fecteau JH, Korjoukov I, Roelfsema PR (2009). Location and color biases have different influences on selective attention. Vision Research.

[CR6] Ferrante O, Patacca A, Di Caro V, Della Libera C, Santandrea E, Chelazzi L (2018). Altering spatial priority maps via statistical learning of target selection and distractor filtering. Cortex.

[CR7] Gao Y, Theeuwes J (2020). Independent effects of statistical learning and top-down attention. Attention, Perception, & Psychophysics.

[CR8] Geng JJ, Behrmann M (2002). Probability cuing of target location facilitates visual search implicitly in normal participants and patients with hemispatial neglect. Psychological Science.

[CR9] Geng JJ, Behrmann M (2005). Spatial probability as attentional cue. Perception & Psychophysics.

[CR10] Geng JJ, Di Quattro NE, Helm J (2017). Distractor probability changes the shape of the attentional template. Journal of Experimental Psychology: Human Perception and Performance.

[CR11] Hoffman ML (1975). Developmental synthesis of affect and cognition and its implications for altruistic motivation. Developmental Psychology.

[CR12] Hoffmann J, Kunde W (1999). Location-specific target expectancies in visual search. Journal of Experimantal Psychology.

[CR13] Huang C, Theeuwes J, Donk M (2021). Statistical learning affects the time courses of salience-driven and goal-driven selection. Journal of Experimental Psychology: Human Perception and Performance.

[CR14] Huang C, Vilotijević A, Theeuwes J, Donk M (2021). Proactive distractor suppression elicited by statistical regularities in visual search. Psychonomic Bulletin & Review.

[CR15] Jiang YV, Swallow KM, Rosenbaum GM, Herzig C (2013). Rapid acquisition but slow extinction of an attentional bias in space. Journal of Experimental Psychology: Human Perception and Performance.

[CR16] Jiang YV, Swallow KM, Won B-Y, Cistera JD, Rosenbaum GM (2015). Task specificity of attention training: The case of probability cuing. Attention, Perception, & Psychophysics.

[CR17] Kuznetsova, A., Brockhoff, P. B., & Christensen, R. H. B. (2017). lmerTest package: Tests in linear mixed effects models. *Journal of Statistical Software, 82*(13), 1–26. 10.18637/jss.v082.i13

[CR18] Lin R, Li X, Wang B, Theeuwes J (2021). Spatial suppression due to statistical learning tracks the estimated spatial probability. Attention, Perception, & Psychophysics.

[CR19] Maljkovic V, Nakayama K (1994). Priming of pop-out: I. Role of features. Memory & Cognition.

[CR20] Miller J (1988). Components of the location probability effect in visual search tasks. Journal of Experimental Psychology: Human Perception and Performance.

[CR21] Posner MI (1980). Orienting of attention. Quarterly Journal of Experimental Psychology.

[CR22] Posner MI, Nissen MJ, Ogden WC, Pick HL, Saltzman E (1978). Attended and unattended processing modes: The role of set for spatial location. *Modes of perceiving and processing information*.

[CR23] Posner MI, Snyder CR, Davidson BJ (1980). Attention and the detection of signals. Journal of Experimental Psychology: General.

[CR24] Ristic J, Kingstone A (2006). Attention to arrows: Pointing to a new direction. Quarterly Journal of Experimental Psychology.

[CR25] Sauter M, Liesefeld HR, Müller HJ (2019). Learning to suppress salient distractors in the target dimension: Region-based inhibition is persistent and transfers to distractors in a nontarget dimension. Journal of Experimental Psychology: Learning, Memory, and Cognition.

[CR26] Shaw ML, Shaw P (1977). Optimal allocation of cognitive resources to spatial locations. Journal of Experimental Psychology: Human Perception and Performance.

[CR27] Stankevich BA, Geng JJ (2014). Reward associations and spatial probabilities produce additive effects on attentional selection. Attention, Perception, & Psychophysics.

[CR28] Theeuwes J (1989). Effects of location and form cuing on the allocation of attention in the visual field. Acta Psychologica.

[CR29] Theeuwes, J. (1991). Cross-dimensional perceptual selectivity Cross-dimensional perceptual selectivity. *Perception & Psychophysics, 50*(2), 184–193. 10.3758/bf03212219 1945740

[CR30] Theeuwes J (1992). Perceptual selectivity for color and form. Perception & Psychophysics.

[CR31] Theeuwes, J. (2018). Visual selection: Usually fast and automatic; seldom slow and volitional. *Journal of*. *Cognition, 1*(1). 10.5334/joc.13 10.5334/joc.13 PMC6634613 31517202

[CR32] Theeuwes J, Van der Burg E (2007). The role of spatial and nonspatial information in visual selection. Journal of Experimental Psychology: Human Perception and Performance.

[CR33] Vecera SP, Rizzo M (2004). What are you looking at? Impaired “social attention” following frontal-lobe damage. Neuropsychologia.

[CR34] Wagenmakers, E. J., Marsman, M., Jamil, T., Ly, A., Verhagen, J., Love, J., Selker, R., Gronau, Q. F., Šmíra, M., Epskamp, S.,Matzke, D., Rouder, J. N., & Morey, R. D (2018). Bayesian inference for psychology. Part I: Theoretical advantages and practical ramifications. *Psychonomic Bulletin and Review, *25(1), 35–57. 10.3758/s13423-017-1343-3 10.3758/s13423-017-1343-3 PMC5862936 28779455

[CR35] Wang, B., & Theeuwes, J. (2018a). Statistical regularities modulate at tentional capture. *Journal of Experimental Psychology: Human Perception and Performance, 44*(1), 13–17. 10.1037/xhp0000472 10.1037/xhp0000472 29309194

[CR36] Wang, B., & Theeuwes, J. (2018b). How to inhibit a distractor location? Statistical learning versus active, top-down suppression. *Attention, Perception, & Psychophysics, 80*(4), 860–870. 10.3758/s13414-018-1493-z 10.3758/s13414-018-1493-z 29476331

[CR37] Wang B, Theeuwes J (2020). Implicit attentional biases in a changing environment. Acta Psychologica.

